# Supplementary material for: Genetic and Pharmacological Manipulations of Glyoxalase 1 Mediate Ethanol Withdrawal Seizure Susceptibility in Mice
Source: Brain Sci. 2021 Jan 19;11(1):127. doi: 10.3390/brainsci11010127 (PMC7835754; doi:10.3390/brainsci11010127)
Supplement: Supplementary file 1 [file brainsci-11-00127-s001.pdf]

Supplemental Table S1: Number of animals per maximum HIC score for each experiment

	Group	No HICs (0)	1	2	3	4
**Experiment 1**	WT	5	3	0	0	0
	TG (B6)	3	4	1	0	0
**Experiment 2**	WT	1	1	6	1	0
	TG (FVB)	0	0	7	3	2
**Experiment 3 (1 cycle)**	Vehicle	7	6	0	0	0
	pBBG	9	2	0	0	0
**Experiment 3 (2 cycles)**	Vehicle	6	7	0	0	0
	pBBG	10	1	0	0	0

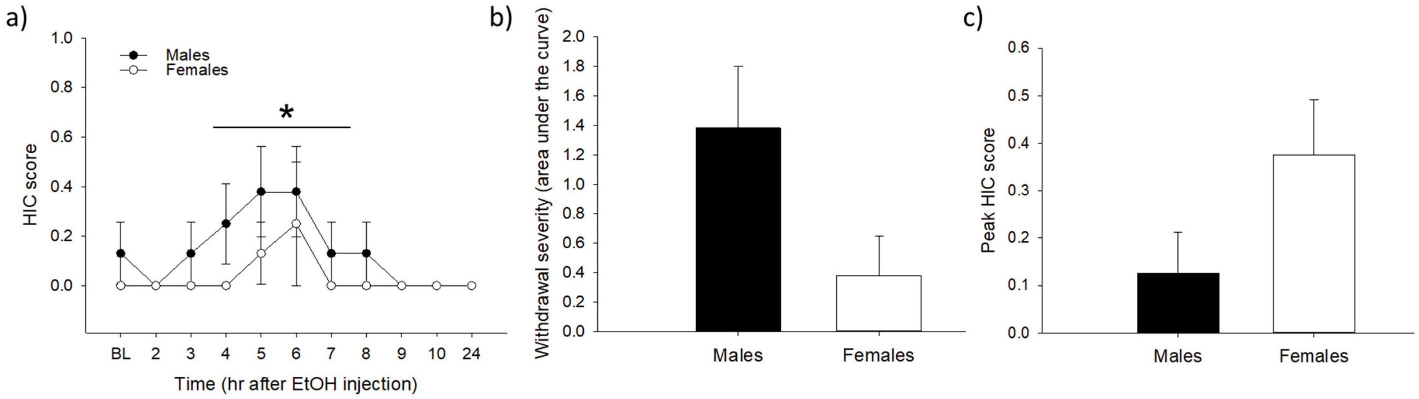

Figure S1. Acute ethanol withdrawal measures for male and female mice from Experiment 1 collapsed on genotype. Panel **a** shows time course of withdrawal, with baseline HIC measurements made immediately before ethanol injection. There was a main effect of sex, with male mice having higher HIC scores across the time course than female mice. Panel **b** shows cumulative HIC scores (area under the curve, AUC) for each sex. There was a non-significant increase in HIC AUC in males compared to females ($p=0.064$). Panel **c** shows peak HIC score for each sex. N=8/genotype. * $p<0.05$
